# Supplementary material for: HIVprotI: an integrated web based platform for prediction and design of HIV proteins inhibitors
Source: J Cheminform. 2018 Mar 9;10:12. doi: 10.1186/s13321-018-0266-y (PMC5845081; doi:10.1186/s13321-018-0266-y)
Supplement: Supplementary file 2 — Additional file 2. Source code of HIVProtI web server. [file 13321_2018_266_MOESM2_ESM.zip › HIVprotI_Source-code/hivprotI/weka-3-6-6/doc/weka/classifiers/mi/package-summary.html]

weka.classifiers.mi


---


|  |  |  |
| --- | --- | --- |
| **Overview** \| **Package** \| Class \| **Tree** \| **Deprecated** \| **Index** \| **Help** \| **Weka's home** | |  |
| **PREV PACKAGE**   **NEXT PACKAGE** | **FRAMES**    **NO FRAMES**     **All Classes** |  |


---

## Package weka.classifiers.mi

| **Class Summary** | |
| --- | --- |
| **CitationKNN** | Modified version of the Citation kNN multi instance classifier.    For more information see:    Jun Wang, Zucker, Jean-Daniel: Solving Multiple-Instance Problem: A Lazy Learning Approach. |
| **MDD** | Modified Diverse Density algorithm, with collective assumption.    More information about DD:    Oded Maron (1998). |
| **MIBoost** | MI AdaBoost method, considers the geometric mean of posterior of instances inside a bag (arithmatic mean of log-posterior) and the expectation for a bag is taken inside the loss function.    For more information about Adaboost, see:    Yoav Freund, Robert E. |
| **MIDD** | Re-implement the Diverse Density algorithm, changes the testing procedure.    Oded Maron (1998). |
| **MIEMDD** | EMDD model builds heavily upon Dietterich's Diverse Density (DD) algorithm.  It is a general framework for MI learning of converting the MI problem to a single-instance setting using EM. |
| **MILR** | Uses either standard or collective multi-instance assumption, but within linear regression. |
| **MINND** | Multiple-Instance Nearest Neighbour with Distribution learner.    It uses gradient descent to find the weight for each dimension of each exeamplar from the starting point of 1.0. |
| **MIOptimalBall** | This classifier tries to find a suitable ball in the multiple-instance space, with a certain data point in the instance space as a ball center. |
| **MISMO** | Implements John Platt's sequential minimal optimization algorithm for training a support vector classifier.    This implementation globally replaces all missing values and transforms nominal attributes into binary ones. |
| **MISVM** | Implements Stuart Andrews' mi\_SVM (Maximum pattern Margin Formulation of MIL). |
| **MIWrapper** | A simple Wrapper method for applying standard propositional learners to multi-instance data.    For more information see:    E. |
| **SimpleMI** | Reduces MI data into mono-instance data. |

---


|  |  |  |
| --- | --- | --- |
| **Overview** \| **Package** \| Class \| **Tree** \| **Deprecated** \| **Index** \| **Help** \| **Weka's home** | |  |
| **PREV PACKAGE**   **NEXT PACKAGE** | **FRAMES**    **NO FRAMES**     **All Classes** |  |


---
